# Clinton County Medical Society

**Published:** 1872-07-15

**Authors:** 


### CLINTON COUNTY MEDICAL SOCIETY.

The Society met at Camanche in regular quarterly session, July 16th, in the office of Dr. Ireland, and at 11 A. M. was called to order by the President, Dr. D. Langan. The Secretary being absent, Prof. P. J. Farnsworth was appointed Secretary, *pro tem.*

The following members responded to the roll call:

| | |
|---|---|
| Dr. D. Langan, | De Witt. |
| " A. W. Morgan, | " |
| " Willis Butterfield, | " |
| " C. H. Lothrop, | Lyons. |
| " G. W. Witherell, | " |
| " P. J. Farnsworth, | Clinton. |
| " H. J. Hobart, | " |
| " A. B. Ireland, | Camanche. |
| " Charles D. Manning, | " |

The minutes of the last meeting read and approved.

The business next in order was the report of Special Committees. The Committee appointed to report to the State Society on a bill for the protection of physicians and surgeons, reported progress, and the Committee, consisting of Drs. Farnsworth, Lothrop and Morgan, was continued.

The Committee on revision of the fee bill reported progress and was continued.

The bill of Dr. Reynold for Secretary's books, seals, &c., was allowed and ordered to be paid. The Society then adjourned till 1½

P. M., and, on invitation of Dr. Ireland, the members repaired to the New Haven House for dinner.

At 1½ P. M. the Society was again called to order and listened to an able essay on Sun-Stroke by Dr. G. W. Witherell, of Lyons. The subject was peculiarly appropriate at this time, on account of the occurrence of so many fatal cases, and the discussion was correspondingly spirited. Dr. C. D. Manning exhibited a pathological specimen—an ulcerated vermiform appendix, obtained by a *post mortem*, made by himself and Dr. Ireland, and Dr. Ireland referred to the great benefits which the community and the profession derive from *post mortem* examinations.

Vice-President Hobart took the Chair while Dr. Langan, delegate to the American Medical Association which met at Philadelphia last May, read a report of the proceedings of the Association, which showed that its last meeting had been one of the largest and pleasantest ever held. On motion of Prof. Farnsworth a vote of thanks was tendered Dr. Langan for representing the Society at the American Medical Association, and it was moved by Dr. Ireland that the report be filed with the Secretary of the Society.

The following gentlemen were appointed to present articles at the next meeting in Lyons:

Dr. A. W. Morgan, on Dislocation and Reduction of the Hip Joint.

Dr. P. J. Farnsworth, Duties of Physicians to each other.

Dr. C. H. Lothrop, Cerebro-Spinal Meningitis.

Dr. Willis Butterfield, New Remedies.

Dr. A. B. Ireland, Obstetrics.

Dr. C. D. Manning, Gelseminum.

On motion thanks were returned to the officers of the C. & N. W. R. R., and to Mr. A. Townley in particular, for accommodations extended to members.

The Society then adjourned, having enjoyed a re-union which keeps the members in fraternal relations with each other, and stimulates them to greater enthusiasm in the profession, while it assists them to exchange facts and observations, and, of course, improvement in medicine affects the public generally. *

